# Transforaminal Epiduroscopic Basivertebral Nerve Laser Ablation for Chronic Low Back Pain Associated with Modic Changes: A Preliminary Open-Label Study

**DOI:** 10.1155/2018/6857983

**Published:** 2018-08-14

**Authors:** Hyeun Sung Kim, Nitin Adsul, Farid Yudoyono, Byapak Paudel, Ki Joon Kim, Sung Ho Choi, Jeong Hoon Kim, Sung Kyun Chung, Jeong-Hoon Choi, Jee-Soo Jang, Il-Tae Jang, Seong-Hoon Oh

**Affiliations:** ^1^Department of Neurosurgery, Nanoori Suwon Hospital, Suwon, Republic of Korea; ^2^Department of Neurosurgery, Hasan Sadikin Hospital, College of Medicine, Universitas Padjadjaran, Bandung, Jawa Barat, Indonesia; ^3^Department of Neurosurgery, Nanoori Hospital, Seoul, Republic of Korea; ^4^Department of Neurosurgery, Nanoori Incheon Hospital, Incheon, Republic of Korea

## Abstract

**Background:**

Chronic low back pain (CLBP) arising from degenerative disc disease continues to be a challenging clinical and diagnostic problem whether treated with nonsurgical, pain intervention, or motion-preserving stabilization and arthrodesis.

**Methods:**

Fourteen patients with CLBP, greater than 6 months, unresponsive to at least 4 months of conservative care were enrolled. All patients were treated successfully following screening using MRI findings of Modic type I or II changes and positive confirmatory provocative discography to determine the affected levels. All patients underwent ablation of the basivertebral nerve (BVN) using 1414 nm Nd:YAG laser-assisted energy guided in a transforaminal epiduroscopic approach. Macnab's criteria and visual analog scale (VAS) score were collected retrospectively at each follow-up interval.

**Results:**

The mean age was 46 ± 9.95 years. The mean symptoms duration was 21.21 ± 21.87 months. The mean follow-up was 15.3 ± 2.67 months. The preoperative VAS score of 7.79 ± 0.97 changed to 1.92 ± 1.38, postoperatively (*P* < 0.01). As per Macnab's criteria, seven patients (50%) had excellent, six patients (42.85%) had good, and one patient (7.14%) had fair outcomes.

**Conclusion:**

The transforaminal epiduroscopic basivertebral nerve laser ablation (TEBLA) appears to be a promising option in carefully selected patients with CLBP associated with the Modic changes.

## 1. Introduction

Chronic low back pain (CLBP) is a serious medico-social problem and a cause of disability. According to the United States National Center for Health Statistics reports, 14% of new hospital visits are patients with chronic low back pain. The expense of treating low back pain is over $100 billion per year [1–3]. In cases with difficulty in identifying the cause of CLBP, magnetic resonance imaging (MRI) can clearly identify pathologies of CLBP. However, the importance of all these pathologies is still controversial [3]. de Roos and coworkers [4] first introduced evidence of the degenerative vertebral endplate and subchondral bone marrow changes due to an inflammatory process of the endplate on MRI. Modic changes were identified in 19%–59% of patients with lumbar degenerative disk disease (DDD) and prolonged back pain. In most cases, nocturnal pain is characteristic, and it causes most commonly localized constant pain [5–8].

The Modic change is closely associated with low back pain [9]. A recent study showed that, in nonspecific low back pain (LBP), the prevalence of any type of Modic change was 46%, as opposed to 6% in the general population [10–13]. Most individuals with Modic changes of more than 90% on MRI will have back pain within one year [8, 13, 14].

**Figure 1:**
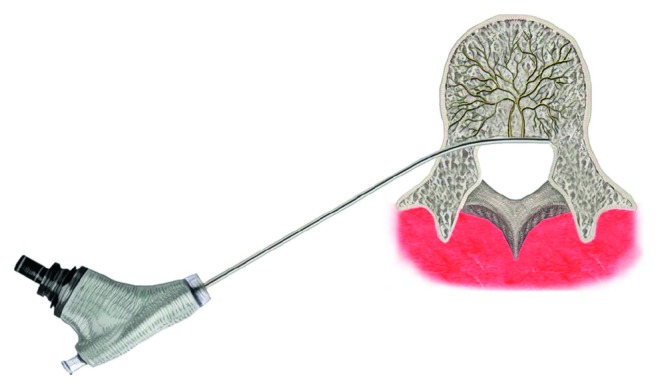
Transforaminal epiduroscopic basivertebral nerve laser ablation (TEBLA) procedure illustration.

Although a few number of patients need surgical interventions, most of them get better spontaneously with conservative management without much worsening in the middle age. Treatment of CLBP associated with Modic changes has not been established yet. We have treated this condition with the transforaminal epiduroscopic approach (Figure 1) with laser targeting to the basivertebral nerve (BVN) with a good outcome. We want to share its efficacy and safety.

## 2. Methods

Patients with isolated CLBP and type 1 or type 2 Modic changes on MRI for more than 6 months' duration that was nonresponsive to at least 3 months of conservative care were analyzed in this single-center retrospective observational study to evaluate the preliminary safety and effectiveness of BVN ablation for the treatment of CLBP with Modic changes.

No specific course of conservative care was mandated before enrollment. Exclusion criteria included were Modic changes without CLBP, spondylolisthesis, scoliosis, history of spinal infection, and prior spinal malignancy. Patients who had radicular symptoms were also excluded. The study was also limited to the L2, L3, L4, L5, and S1 vertebrae. Patients with the identified pathology at more cephalad levels were excluded. Determination of the treatment level(s) was made by clear evidence of Modic type 1 or type 2 changes on MRI and by provocative discography.

Treatment was limited to two or three contiguous vertebral bodies representing one or two motion segments, respectively. A detailed history and physical examination were performed on each patient to exclude origins of pain such as sacroiliac joint, hip, myofascial, genitourinary, gastrointestinal, or gynecologic sources.

We performed 3 steps of provocation tests to check the Modic changes as the source of pain as follows: (1) preoperative provocative discography, (2) intraoperative laser provocation into the basivertebral nerve of the vertebral body with Modic changes, and (3) intraoperative relief of pain assessment by laser reprovocation of the same painful area after completion of the laser ablation procedure.

### 2.1. Surgical Technique

Fluoroscopic guidance during procedures in the anteroposterior and lateral projection was essential to ensure a precise skin entry point. Anesthesia was limited to 1% local lidocaine 7–10 cc with epinephrine mixed with 1.6% lidocaine 2-3 cc, 3–5 minutes after the 1st injection. In the provocation steps, the main painful point was commonly located in the upper endplate of the lower vertebral body. The needle was inserted into the suprapedicular notch [15] to get access to the vertebral endplate with the Modic change through the epidural space near to the upper endplate of the lower vertebral body. A guide wire was inserted through the needle into the suprapedicular notch. After withdrawing the needle, a tapered cannulated obturator was slid over the guide wire and advanced into the epidural space. A beveled working cannula was introduced over the obturator, and the obturator was removed. Semirigid epiduroscope, NeedleView CH (Lutronic®, Ilsan, South Korea) (Figure 1), was introduced through the working cannula. For laser ablation, a side-firing Nd : YAG laser cable included in the TELA (transforaminal epiduroscopic laser ablation) system (Lutronic®, Ilsan, South Korea) with a wavelength of 1414 nm operating in the 0.75–12 watt range was used. However, for our patients, we used a range of 4.5∼6 W. This laser system is capable of thermovibration and can ablate an approximately spherical region of 10 mm diameter (5 mm radius). The laser cable was guided to the predetermined target (BVN terminus) near the Modic change area under fluoroscopic imaging. Each vertebral endplate having a painful Modic change was treated giving a laser locally at the BVN terminus by a 1414 nm Nd : YAG laser system (Figure 1). Self-reported outcome instruments were used to assess the patient's condition preoperatively and at each follow-up. VAS score and Macnab's criteria were used in this study. Neurologic examination and adverse event screening and reporting were also performed at each follow-up.

The clinical outcomes were evaluated using descriptive statistical analysis. We conducted Student's paired *t*-test to evaluate the significant difference in the preoperative and postoperative clinical outcome parameter, that is, VAS score. The significance level was considered at 0.05. The statistical software used for the study was SPSS version 20.0 (IBM Corp., Armonk, New York, USA).

## 3. Results

**Figure 2:**
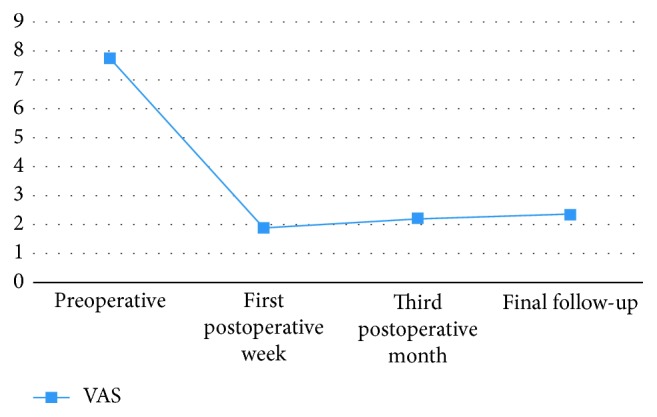
Graphs showing changes in the visual analog scale (VAS) score.

A total of 14 consecutive patients were analyzed. The mean age was 46 ± 9.95 (range: 31–63) years. There were six men and eight women; in all patients, MRI findings correlated with clinical findings. The mean symptoms duration was 21.21 ± 21.87 (range: 4–84) months. The mean follow-up was 15.3 ± 2.67 months (range: 12–20 months). Pfirmann's disc degeneration grade was grade IV in 8 (57.14%) patients and grade V in 6 (42.85%) patients. Eight and six patients had Modic type 1 and type 2 changes, respectively (Table 1). The preoperative VAS score was 7.79 ± 0.98 (range: 6–10), and the postoperative VAS score improved to 1.93 ± 1.39 (range: 1–6). The improvement was significant at *P* < 0.0001 (Table 2). Significant improvement was maintained at the third month and at final follow-up with VAS scores of 2.21 ± 0.89 (range: 1–5; *P* < 0.0001) and 2.36 ± 1.01 (range: 1–5; *P* < 0.0001), respectively (Figure 2). As per Macnab's criteria, seven patients (50%) had excellent, six patients (42.85%) had good, and one patient (7.14%) had fair outcomes (Table 3).

There were no occurrences of infections, discitis, paresis, dural tears, vascular injuries, or systemic complications until the latest follow-up. Furthermore, the patient's VAS scores for back pain and Macnab's criteria were maintained at the final follow-up compared with postoperative scores. All patients maintained their functional neurologic status throughout the entire study follow-up. There were no device- or procedure-related serious adverse events in this study.

## 4. Discussion

**Figure 3:**
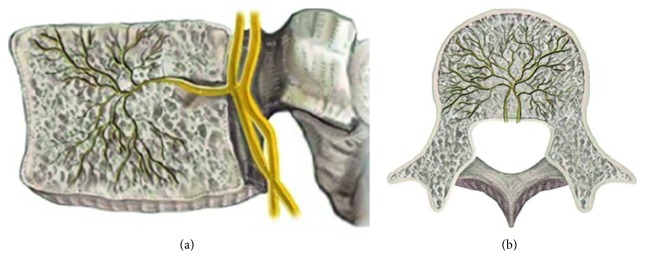
Anatomy of the basivertebral nerve. (a) Sagittal view. (b) Axial view.

**Figure 4:**
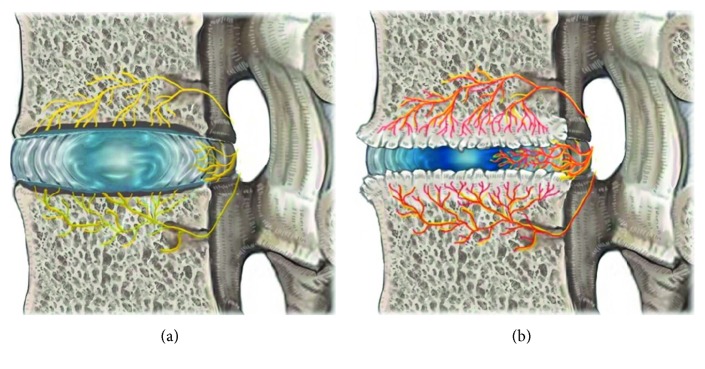
(a) Anatomy of BVN with a sinuvertebral nerve in the normal disc. (b) Pathological disc.

The Modic change caused by disc degenerative processes involving damage and microfractures to the vertebral endplates results in the development of edema and inflammation of the adjacent vertebrae [5, 11]. Factors associated with endplate damage following degenerative disc disease, ingrowth of new vessels, and collection of inflammatory agents may be important characteristics in the generation of pain [5, 6, 11, 16]. In the normal state, bony endplates have a rich supply of small free nerve endings; degeneration and inflammation will likely trigger the inflammatory pain [13] (Figures 3 and 4).

Modic change is associated with chronic back pain [8]. While previous studies concluded that, among Modic changes, type 1 changes are the strongest and most crucial element in the disc degenerative process in relation to LBP, they also demonstrated that DDD with Modic changes was much more frequently associated with clinical symptoms [6, 11, 16]. Our patients also had mainly Modic type 1 changes, but some patients with Modic type 2 changes were also symptomatic in provocative discography; so they were also treated with TEBLA.

A study by Becker and coworkers reported that the primary innervation for the vertebral body is by the BVN that enters the bone of the vertebral body via the midline of the posterior cortex. The large, usually paired, neurovascular foramina are located equidistant from each endplate. These nerves accompany the basivertebral artery and vein [17] (Figure 3). Previous studies also reported on the innervation within the bone marrow with the presence of immunoreactive neural tissue in rat vertebral bodies and histologically identified intraosseous nerves and neurovascular bundles.

**Figure 5:**
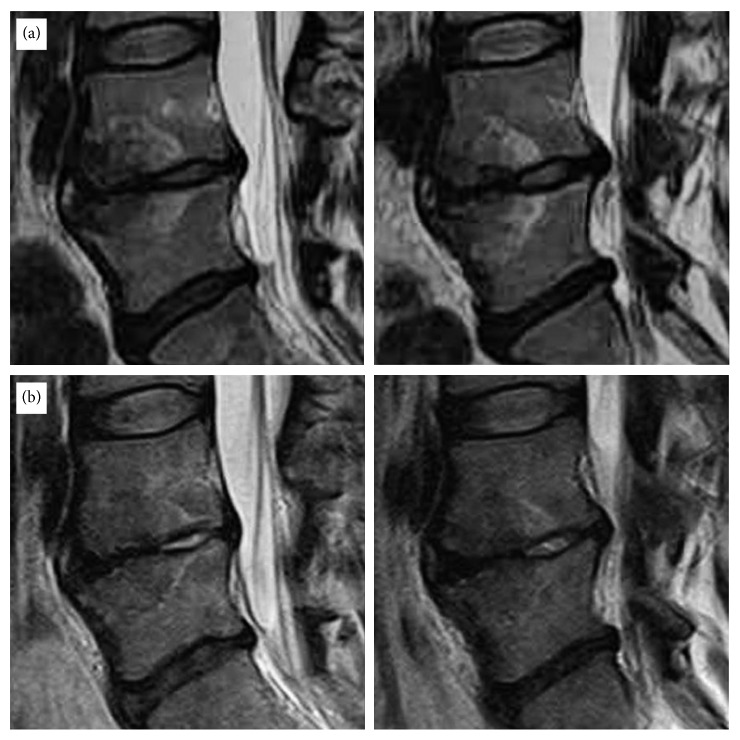
Magnetic resonance imaging (MRI) of a 35-year-old female patient who had suffered severe back pain and bilateral referred leg pain for more than 2 years. This patient's symptom improved significantly after TEBLA at the L4-5 level. The preoperative VAS score of 8 decreased to 1 postoperatively. (a) Preoperative T2WI lumbar magnetic resonance imaging (MRI) sagittal views with Modic Type 1 changes. (b) Postoperative T2WI lumbar MRI sagittal views showing resolution of the Modic reaction.

**Figure 6:**
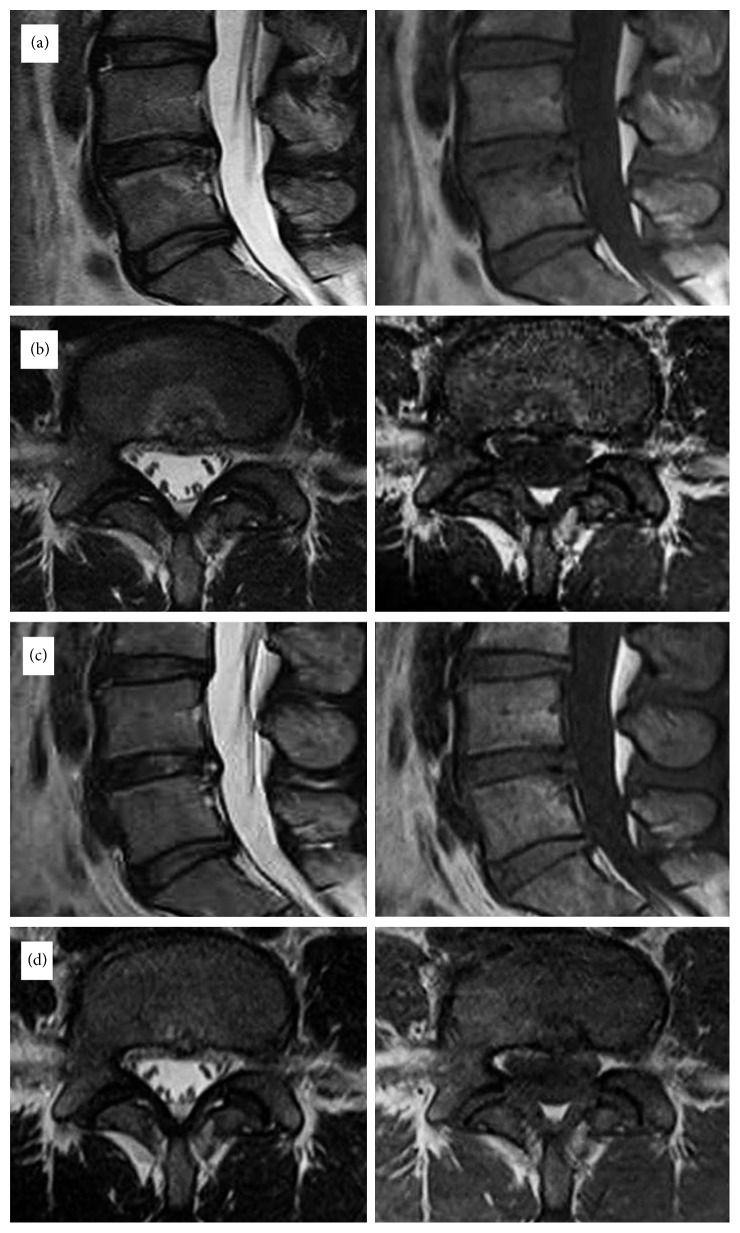
Magnetic resonance imaging (MRI) of a 31-year-old female patient who had suffered severe back pain and bilateral referred leg pain for more than 2 years. This patient's symptom improved significantly after TEBLA at the L4-L5 level. The preoperative VAS score of 8 decreased to 1 postoperatively. Preoperative T1WI and T2WI lumbar magnetic resonance imaging (MRI) sagittal (a) and axial (b) views with Modic type 1 changes. Postoperative T1WI and T2WI lumbar MRI sagittal (c) and axial (d) views showing resolution of the Modic reaction.

**Figure 7:**
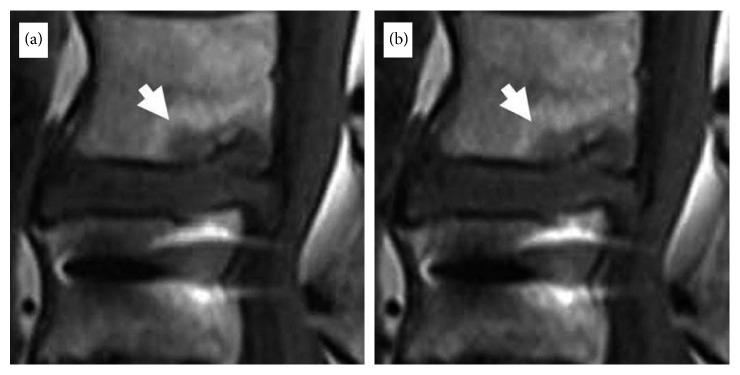
Magnetic resonance imaging (MRI) of a 65-year-old male patient who had fusion surgery previously and was suffering from back pain and buttock pain for more than 28 months. The preoperative VAS score of 7 decreased to 1 postoperatively. (a) Preoperative T1WI lumbar magnetic resonance imaging (MRI) sagittal view with Modic type 1 changes. (b) T1WI lumbar MRI taken after 6 months of the TEBLA sagittal view showing resolution of the Modic reaction (white arrow).

Basivertebral nerve sensitization causes CLBP. Substance P, a peptide neurotransmitter of the tachykinin family, is found within the basivertebral nerve, and there is very strong evidence that this nerve has the potential for transmitting pain signals [16, 18–20]. Ablation of this nerve was our target. A recent study also supports ablation of the basivertebral nerve for CLBP associated with Modic changes. They used radiofrequency through the transpedicular and extrapedicular approach and obtained significant improvement in ODI [17]. Our study differs from this study. We used the transforaminal epiduroscopic approach (Figure 1) with three steps of provocation. We used a laser instead of radiofrequency. Some of the representative cases are illustrated in Figures 5–7.

The major limitation of this study is the lack of a comparative control group. Similar to any other retrospective studies, limitations associated with our study include retrospective clinical outcome assessments and small sample size since very few patients actually require surgical intervention. Nevertheless, after TEBLA, we got 93% good-to-excellent results according to Macnab's criteria, and the preoperative VAS score of 7.79 ± 0.97 decreased to 1.92 ± 1.38 at the latest follow-up (*P* < 0.0001). This result is encouraging and the procedure was safe.

## 5. Conclusion

The TEBLA appears to be a promising option in carefully selected patients with chronic low back pain associated with the Modic changes. The treatment improved patients' self-reported outcomes, and the improvement was largely maintained through more than 12 months of the follow-up period.

## Figures and Tables

**Table 1:** Patient characteristics of TEBLA.

Characteristic	Value
Patients (*n*)	14
Sex (*n*)	Women = 8/men = 6
Age (years)	46 ± 9.95 (31−63)
Symptoms duration (months)	21.21 ± 21.87 (4−84)
Follow-up duration (months)	15.3 ± 2.67 (12−20)
*Surgical level*	
L2-3	3
L3-4	2
L4-5	1
L4-5-S1	1
L5-S1	7
Number of complications	0
*Macnab criteria*	
Excellent	7 (50%)
Good	6 (43%)
Fair	1 (7%)
Poor	0

**Table 2:** Clinical outcomes.

Score	Preoperative^ǂ^	Postoperative^ǂ^	*P* value^*∗*^	3-month postoperative^ǂ^	*P* value^*∗*^	Final follow-up^ǂ^	Value^*∗*^
VAS score	7.79 ± 0.98	1.93 ± 1.39	<0.0001	2.21 ± 0.89	<0.0001	2.36 ± 1.01	<0.0001

^*∗*^For statistical analysis, the paired *t*-test was used. *P* value <0.05 was considered significant. ^ǂ^All values are expressed as mean ± standard deviation.

**Table 3:** Individual patient details with the pain response.

Number	Age	Sex	Symptom duration (months)	Level	VAS score				ODI				Macnab's criteria
					Pre-op	Post-op (1 week)	Post-op (3 months)	Final follow-up	Pre-op	Post-op (1 week)	Post-op (3 months)	Final follow-up	Post-op (1 week)
1	45	F	15	L5-S1	7	1	2	2	46	24	22	24	Good
2	56	M	8	L5-S1	7	1	1	1	52	18	19	18	Excellent
3	41	M	7	L3-4	8	2	2	2	64	24	22	24	Excellent
4	50	F	6	L2-3	8	1	2	1	58	22	26	24	Excellent
5	44	F	24	L5-S1	8	2	2	3	61	24	26	28	Excellent
6	62	M	28	L2-3	8	1	2	2	65	18	20	22	Excellent
7	37	F	6	L3-4	9	1	2	3	68	20	22	25	Good
8	35	F	24	L5-S1	8	1	3	2	58	22	26	24	Excellent
9	55	F	84	L5-S1	8	2	2	3	56	24	23	27	Good
10	31	F	24	L5-S1	8	1	2	2	63	18	21	22	Excellent
11	63	M	48	L2-3	6	6	5	5	54	48	42	42	Fair
12	41	M	5	L5-S1	10	3	2	3	72	28	24	24	Good
13	38	F	14	L4-5	7	3	2	2	55	28	25	25	Good
14	46	M	4	L4-5-S1	7	2	2	2	58	23	23	24	Good

## References

[1] Zhang Y.-g., Guo T.-m., Guo X., Wu S.-x. (2009). Clinical diagnosis for discogenic low back pain. *International Journal of Biological Sciences*.

[2] Peng B.-G. (2013). Pathophysiology, diagnosis, and treatment of discogenic low back pain. *World Journal of Orthopedics*.

[3] Tonosu J., Inanami H., Oka H. (2016). Diagnosing discogenic low back pain associated with degenerative disc disease using a medical interview. *PLoS One*.

[4] de Roos A., Kressel H., Spritzer C., Dalinka M. (1987). MR imaging of marrow changes adjacent to end plates in degenerative lumbar disk disease. *American Journal of Roentgenology*.

[5] Albert H. B., Kjaer P., Jensen T. S., Sorensen J. S., Bendix T., Manniche C. (2008). Modic changes, possible causes and relation to low back pain. *Medical Hypotheses*.

[6] Rahme R., Moussa R. (2008). The modic vertebral endplate and marrow changes: pathologic significance and relation to low back pain and segmental instability of the lumbar spine. *American Journal of Neuroradiology*.

[7] Manniche C. (2014). Vertebral endplate (modic) changes and the treatment of back pain using antibiotics. *Clinical Practice*.

[8] Jensen R. K., Kent P., Hancock M. (2015). Do MRI findings identify patients with chronic low back pain and Modic changes who respond best to rest or exercise: a subgroup analysis of a randomised controlled trial. *Chiropractic and Manual Therapies*.

[9] Mok F. P., Samartzis D., Karppinen J., Fong D. Y., Luk K. D., Cheung K. M. (2016). Modic changes of the lumbar spine: prevalence, risk factors, and association with disc degeneration and low back pain in a large-scale population-based cohort. *Spine Journal*.

[10] Braithwaite I., White J., Saifuddin A., Renton P., Taylor B. (1998). Vertebral end-plate (Modic) changes on lumbar spine MRI: correlation with pain reproduction at lumbar discography. *European Spine Journal*.

[11] Albert H. B., Sorensen J. S., Christensen B. S., Manniche C. (2013). Antibiotic treatment in patients with chronic low back pain and vertebral bone edema (Modic type 1 changes): a double-blind randomized clinical controlled trial of efficacy. *European Spine Journal*.

[12] Sheng-yun L., Letu S., Jian C. (2014). Comparison of modic changes in the lumbar and cervical spine, in 3167 patients with and without spinal pain. *PLoS One*.

[13] Manniche C, Jordan A (2016). 10 years of research: from ignoring Modic changes to considerations regarding treatment and prevention of low-grade disc infections. *Future Science OA*.

[14] Eser O., Gomleksiz C., Sasani M. (2013). Dynamic stabilisation in the treatment of degenerative disc disease with modic changes. *Advances in Orthopedics*.

[15] Kim H. S., Ju C. I., Kim S. W., Kim J. G. (2009). Endoscopic transforaminal suprapedicular approach in high grade inferior migrated lumbar disc herniation. *Journal of Korean Neurosurgical Society*.

[16] Heggeness M., Becker S., Hadjipavlou A. (2011). Ablation of the basivertebral nerve for the treatment of back pain: a pilot clinical study. *Spine Journal*.

[17] Becker S., Hadjipavlou A., Heggeness M. H. (2017). Ablation of the basivertebral nerve for treatment of back pain: a clinical study. *Spine Journal*.

[18] Hirsh C. (1963). The anatomical basis for low back pain: studies on the presence of sensory nerve endings in ligamentous, capsular and intervertebral disc structures in the human lumbar spine. *Acta Orthopaedica Scandinavica*.

[19] Antonacci M. D., Mody D. R., Heggeness M. H. (1998). Innervation of the human vertebral body: a histologic study. *Journal of Spinal Disorders*.

[20] Fras C., Kravetz P., Mody D. R., Heggeness M. H. (2003). Substance P-containing nerves within the human vertebral body: an immunohistochemical study of the basivertebral nerve. *Spine Journal*.

